# Research on HTLV-1 and HTLV-2 in Latin America and the Caribbean over the last ten years

**DOI:** 10.1016/j.heliyon.2023.e13800

**Published:** 2023-02-15

**Authors:** Dustin M. Solorzano-Salazar, Akram Hernández-Vásquez, Fabriccio J. Visconti-Lopez, Diego Azañedo

**Affiliations:** a Universidad Peruana de Ciencias Aplicadas, Lima, Peru; b Centro de Excelencia en Investigaciones Económicas y Sociales en Salud, Vicerrectorado de Investigación, Universidad San Ignacio de Loyola, Lima, Peru; c Independent Researcher, Lima, Peru; d Universidad Científica del Sur, Lima, Peru

**Keywords:** Human *t*-lymphotropic virus, HTLV, Caribbean region, Latin America, Bibliometrics, Trends

## Abstract

Worldwide, Human T-lymphtropic virus-1 and 2 (HTLV-1 and 2) infects approximately more than 10 million people, mostly occurring in hyperendemic areas such as the region of Latin America and the Caribbean (LAC). A comprehensive bibliographic exploration of original articles published on the Web of Science Core Collection database over the last 10 years was done. A bibliometric analysis was performed using the bibliometrix package in RStudio and VOSviewer. A total of 519 articles published in 194 journals were identified along the 10 years studied. In 2012 the peak number of publications was identified and the average number of citations per document was 1.33. Galvao-Castro B was the author with the greatest number of publications. *Aids Research and Human Retroviruses* was the most productive journal, and the study by Bangham CRM was the most cited. Brazil was the country with most corresponding authors that had the most publications and the most significant number of total citations. Infections and HTLV-1 were the most used keywords. In conclusion, according to the current quantitative analysis, there is a need for more significant promotion of research on HTLV-1 and 2 among the scientific community of LAC.

## 1 Introduction

Worldwide, it is estimated that the number of cases of Human T-lymphtropic virus infection is of approximately 10 million for type 1 (HTLV-1) and 800 thousand for type 2 (HTLV-2) [1,2]. The epidemiological distribution of these viruses is particularly heterogeneous, with areas of hyperendemicity existing in indigenous communities compared to urban communities [1,[3], [4], [5]]. This occurs especially in the countries of Latin America and the Caribbean (LAC), West Africa and Southwest Japan, which are the regions with the highest prevalence of infection by these viruses in the world [4,6]. Research on the burden and impact of the infection by these viruses is important in these endemic regions in order to implement cost-effective preventive measures to control their spread and complications.

HTLV-1 and HTLV-2 are retroviruses that are associated with a number of acute, chronic, and inflammatory disorders [7]. HTLV-1 infection is associated with life-threatening diseases such as adult T-cell leukemia and HTLV-1 associated myelopathy (HAM) [8]. On the other hand, no specific disease caused by HTLV-2 infection has been demonstrated [7]. However, individuals infected with HTLV-1 and HTLV-2 present physical problems, such as weakness and chronic pain, and socio-psychological problems, such as stigmatization and depression due to being a carrier of HTLV-1 and HTLV-2 [9,10]. Likewise, infection by these viruses exacerbates other diseases such as tuberculosis and strongyloidiasis, which are highly prevalent in the LAC region, increasing the complexity and cost of their management [11]. On the other hand, no effective treatment for HTLV-1 and HTLV-2 infection is currently available, making the prevention and control of the spread of these viruses essential to reduce the burden of disease in the population, particularly in endemic areas in LAC [9].

The LAC region presents sociodemographic characteristics that could explain the endemicity of HTLV-1 and HTLV-2 compared to other regions. Low socioeconomic and educational levels, as well as high rates of sexual risk behaviors and intravenous drug abuse may be the main reasons for the high burden of this disease in this region [[12], [13], [14]]. Moreover, the lack of resources for HTLV-1 screening in lactating mothers and blood donors generates another public health challenge in LAC [15]. Therefore, it is important not to neglect the endemicity of HTLV-1 in LAC, since prevention measures in other countries have managed to reduce the prevalence of these viruses [[13], [14], [15], [16], [17]].

A bibliometric study allows a quantitative analysis to be carried out to evaluate the development of the publications made on certain topics in established periods of time [18]. Compared to narrative studies, bibliometric studies integrate quantitative and statistical analyses as part of their methodology, making them less susceptible to researcher bias [19]. Although it is estimated that the scientific production of HTLV-1 and HTLV-2 worldwide has remained constant in recent years, there is no updated article at the global level that describes if the scientific production of this disease has changed in other regions, especially in endemic regions such as LAC [20]. Likewise, knowing the state of research trends on HTLV-1 and HTLV-2 over time will put into perspective the areas of interest in scientific productivity on this topic in LAC, which may help guide future research. Therefore, the objective of this study was to determine the bibliometric characteristics of scientific articles about HTLV-1 and HTLV-2 in LAC.

## 2 Materials and methods

### 2.1 Data source and search strategy

Web of Science (WOS) Core Collection is one of the largest and most important databases on various fields of knowledge and is widely used in studies to measure scientific output [21,22]. The Journal Impact Factor was used to obtain the impact factor and the quartile of a journal category according to the Journal Citation Reports.

One author developed the search strategy, which was validated by the remaining authors. The full search strategy is described in Supplementary Material. The search was carried out on August 11, 2022. The types of documents were limited to original articles published in the last 10 years (2012–2021).

### 2.2 Data collection

The complete records of the search results were exported in.ciw format and then imported into Rayyan [23]. One of the authors reviewed each abstract title to assess compliance with the inclusion criteria which were the following: 1) the article deals with HTLV-1 and/or HTLV-2; 2) was carried out by at least one author or in a population of one of the Latin American countries according to the SJR classification (https://www.scimagojr.com/countryrank.php?region=Latin%20America). Finally, the complete records and cited references of all Accession Number codes of the articles included in the WOS were obtained (see list of codes in Supplementary Material).

Author names, organizations, and keywords were manually standardized by one author to correct and/or group similarities in order to obtain a plain text file (.txt) for analysis in VOSviewer [24].

### 2.3 Data analysis

The data were analyzed and visualized using Excel, bibliometrix [25], Biblioshiny, and VOSviewer [24]. The frequencies of the articles, the most productive authors, the most frequent KeyWords Plus, the scientific production of the countries, and the most cited articles were calculated using the bibliometrix package (version 4.0.0) in the R software (version 4.2.0). Biblioshiny was used to obtain a map of collaborations between countries. The analysis in VOSviewer included a network analysis for authors, organizations, and keywords (All Keywords: includes Authors keywords and KeyWords Plus) using a Full counting method, a threshold of 5 and a maximum number of 25 authors or countries per document were selected.

### 2.4 Ethical considerations

Since all the data used here were obtained from a bibliographic database, no ethical review was required.

## 3 Results

**Fig. 1:**
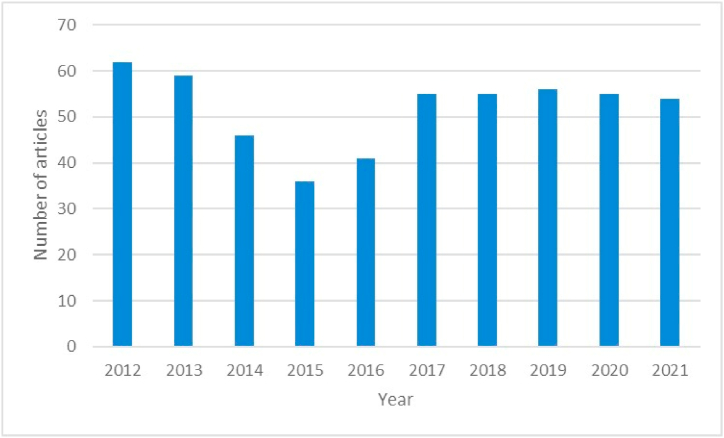
Evolution of the publication of articles on HTLV in the Web of Science.

A total of 519 articles on HTLV-1 and HTLV-2 in LAC were included in the WOS and published in 194 journals. During the study period, the average number of annual publications was 5.47, with a maximum number of 62 articles published in 2012 and a minimum of 36 articles published in 2015. The average number of citations per document per year was 1.33 (Fig. 1).

**Fig. 2:**
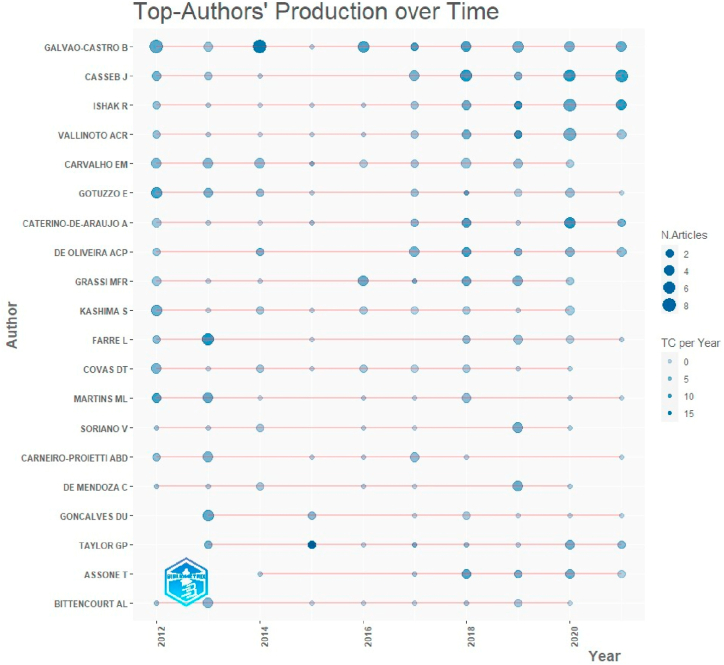
Scientific production of the top authors over time. TC: total citations.

**Fig. 3:**
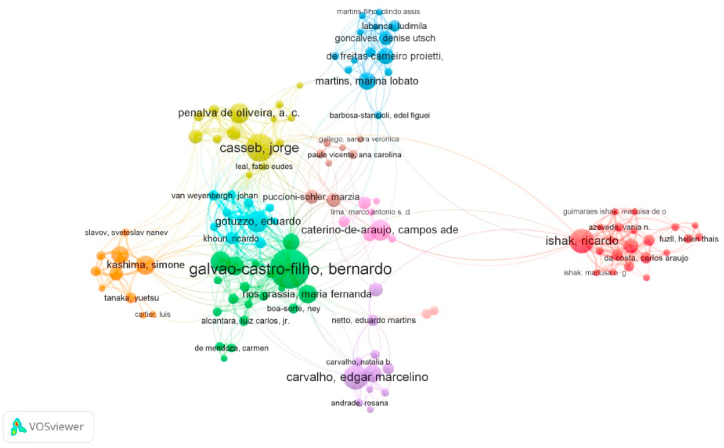
Network analysis of co-authorship of articles on HTLV.

Among the 2442 authors found, the authors with the highest productivity were Galvao-Castro B, Casseb J, and Ishak R, Vallinoto ACR, and Carvalho EM, with 44, 34, 24, 22, and 20 articles, respectively. In addition, in 2014, Galvao-Castro B had the highest number of publications made in one year (8 articles) (Fig. 2, Fig. 3). It is worth mentioning that among the 10 most productive authors, 9 were Brazilian and 1 Peruvian.

The Bangham CRM study was the most cited within the study period, with a total of 119 citations and an average of 14.9 citations per year from 2015 to 2021. In second and third place are Polat M (2016) and Carcamo CP (2012), with a total of 71 and 65 citations and 10.1 and 5.9 citations per year, respectively. On the other hand, the most recent article to enter the top 10 most cited articles was published in 2018 by Malpica L, who has 47 publications and an average of 9.4 citations per year (Table 1).

**Table 1:** Top 10 most cited articles on HTLV in LAC.

N	Article	DOI	Local Citations	Global Citations	Normalized Local Citations	Normalized Global Citations	TC per Year
1	Bangham CRM, 2015, Nat Rev Dis Primers [26]	10.1038/nrdp.2015.12	30	119.0	7.40	8.81	14.9
2	Polat M, 2016, Retrovirology [27]	10.1186/s12977-016-0239-z	0	71.0	0.00	6.63	10.1
3	Carcamo CP, 2012, Lancet Infect Dis [28]	10.1016/S1473-3099(12)70,144-5	3	65.0	0.61	3.88	5.9
4	Gessain A, 2012, Rev Neurol-France [29]	10.1016/j.neurol.2011.12.006	17	59.0	3.44	3.52	5.4
5	Einsiedel L, 2012, Clin Infect Dis [30]	10.1093/cid/cir766	4	58.0	0.81	3.46	5.3
6	Furtado MDBS, 2012, J Med Virol [31]	10.1002/jmv.23,227	26	55.0	5.27	3.29	5
7	Tanajura D, 2015, Clin Infect Dis [32]	10.1093/cid/civ229	23	53.0	5.67	3.93	6.6
8	Gillet Na, 2013, Plos Pathog [33]	10.1371/journal.ppat.1,003,687	0	51.0	0.00	4.58	5.1
9	Malpica L, 2018, Blood Adv [34]	10.1182/bloodadvances.2,017,011,106	3	47.0	1.39	5.76	9.4
10	Gillet Na, 2013, Plos Pathog [35]	10.1371/journal.ppat.1,003,263	0	47.0	0.00	4.22	4.7

TC: total citations.

The journals with the most publications with an author from LAC were *Aids Research and Human Retroviruses* (29 articles; United States), *Revista da Sociedade Brasileira de Medicina Tropical* (29 articles; Brazil), and *Plos Neglected Tropical Diseases* (28 articles; United States). In addition, among the 10 journals with the highest number of publications, 5 were from the United States, 2 from Brazil, 2 from the United Kingdom and 1 from the Netherlands (Table 2).

**Table 2:** Top 10 journals with the highest number of publications.

N	Journal	Country	n	IF	Quartile
1	AIDS Research and Human Retroviruses	United States	29	1.723	Q4
2	Revista da Sociedade Brasileira de Medicina Tropical	Brazil	29	2.141	Q3
3	Plos Neglected Tropical Diseases	United States	28	4.781	Q1
4	Plos One	United States	27	3.752	Q2
5	Brazilian Journal of Infectious Diseases	Brazil	23	3.257	Q3
6	Journal of Medical Virology	United States	20	20.693	Q1
7	BMC Infectious Diseases	United Kingdom	12	3.667	Q3
8	Journal of Neurovirology	United Kingdom	12	3.739	Q3
9	American Journal of Tropical Medicine And Hygiene	United States	11	3.707	Q2
10	International Journal of Infectious Diseases	Netherlands	11	12.074	Q1

IF: impact factor. n: number. Q: quartile. Source of IF and Quartile: Journal Citation Reports™ 2021.

The country of corresponding authors with the highest number of articles on HTLV in LAC was Brazil, with 313 articles, representing 60% of all publications. The second place was taken by the United States, with 43 articles, representing 8% of the total publications. Likewise, the largest number of single country publications (SCP) was in Brazil, with 249/519 (48.0%). Similarly, the country with the highest number of citations was Brazil, with 2552 citations and an average of 8.2 citations per article; followed by the United States, with 612 citations and an average of 14.3 citations per article (Table 3).

**Table 3:** Top 10 countries of authors with publications on HTLV.

N	Country	n	%	SCP	MCP	Total citations	Average article citations
1	Brazil	313	60.1	249	64	2552	8.2
2	United States	43	8.3	23	20	612	14.3
3	France	23	4.4	12	11	232	10.1
4	Argentina	18	3.5	14	4	83	4.6
5	Peru	16	3.1	7	9	68	4.3
6	Spain	16	3.1	10	6	123	7.7
7	Chile	13	2.5	7	6	96	7.4
8	United Kingdom	12	2.3	6	6	291	24.5
9	Colombia	10	1.9	9	1	34	3.4
10	Belgium	8	1.5	0	8	125	15.6

SCP: single country publications. MCP: multiple country publications; n: number.

**Fig. 4:**
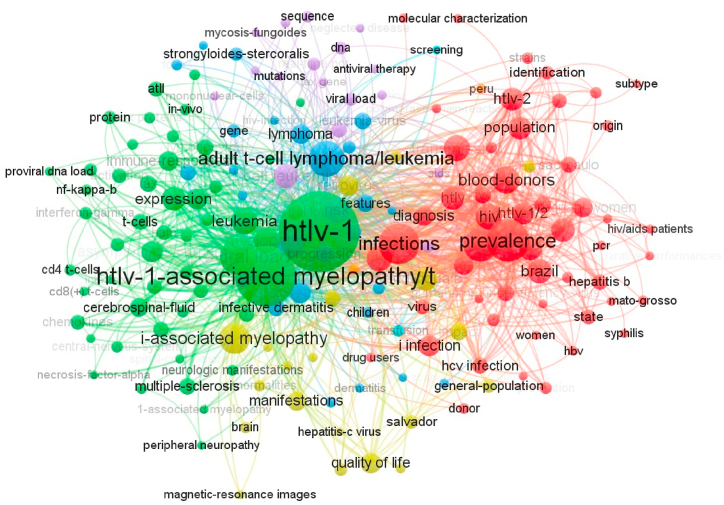
Network analysis of co-occurrence of terms.

**Fig. 5:**
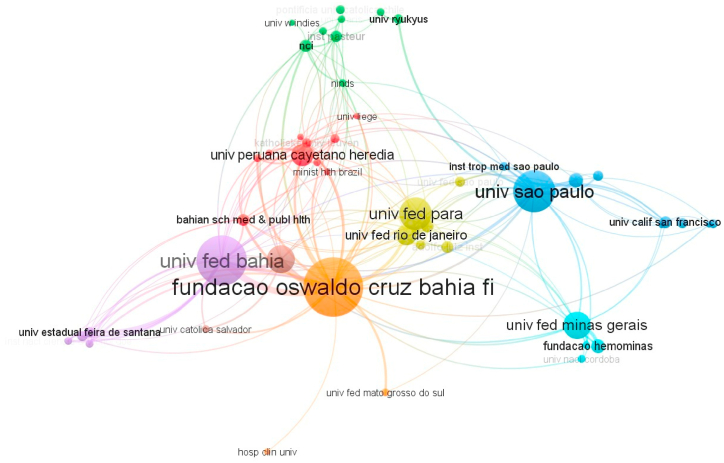
Network analysis of organization co-authorship.

**Fig. 6:**
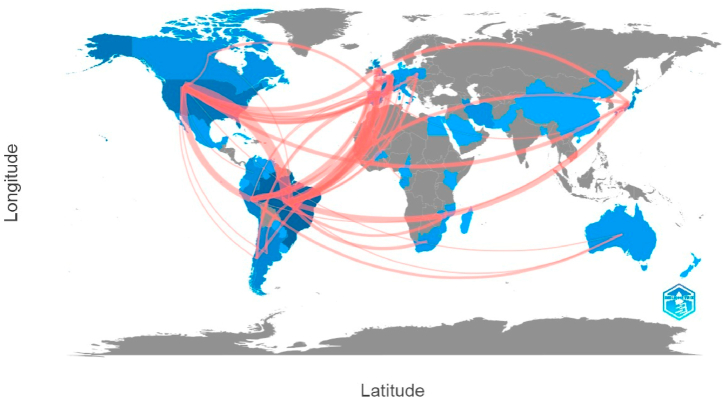
Country collaboration map.

Of the total of 1095 Keywords Plus, the most used were HTLV-1, followed by HTLV-1-associated myelopathy/tropical spastic paraparesis, and infections and prevalence (Fig. 4). Finally, the institutional affiliations with the highest number of articles published on HTLV in LAC were: *Fundação Oswaldo Cruz Bahia (FIOCRUZ-Bahia)*, University of *São Paulo*, Federal University of *Pará*, and Federal University of *Bahia* (Fig. 5). We must remark that the five most cited authors (Fig. 2) correspond to this list of affiliations, being Galvao-Castro B from FIOCRUZ-Bahia, Casseb J from the University of *Sao Paulo*, Ishak R and Vallinoto ACR from the Federal University of *Pará*, and Carvalho EM from the Federal University of *Bahia*. Regarding the analysis of collaborative networks by country, within the LAC countries, Brazil, Peru, and Argentina had the greatest number of collaborations with the majority of LAC and non-LAC countries. Furthermore, the analysis revealed a well-established collaboration network between Brazil and the United States (Fig. 6).

## 4 Discussion

The present study determined the bibliometric characteristics of the scientific production of HTLV-1 and HTLV-2 between 2012 and 2021 using the WOS database. A total of 519 publications in 194 journals were explored, reporting an average of 5.47 annual publications. The study most cited during the last 10 years was that of Bangham CRM (2015) [26]. Similarly, the author with the most publications was Galvao-Castro B (44 articles). The journals with the most publications were *AIDS Research and Human Retrovirus* and the *Revista da Sociedade Brasileira de Medicina Tropical*. Likewise, the corresponding authors with the highest number of publications belonged to institutions in Brazil and the United States. The most used keywords were “HTLV-1” and “HTLV-1-associated myelopathy/tropical spastic paraparesis”, “Infections” and “Prevalence”.

According to our analysis, during the study period there was a decrease in the trend of annual publications on HTLV-1 and HTLV-2 between 2012 and 2015; with a slight growth towards 2017, which has remained stationary until 2021. Previously, a reduction in scientific productivity on HTLV-1 was reported around the world [36], with some relative growth by LAC researchers [20]. Despite the COVID-19 pandemic complicating the surveillance of some diseases and the development of research in recent years, especially in relation to neglected diseases [37,38], our analysis did not show a substantial variation in the number of articles produced during 2020–2021. However, scientific production remains modest and without great advances in the treatment of viral infection [9].

In relation to the most productive authors during the 2012–2021 period, the first three were Brazilian. In first place, the author Galvao-Castro B, a professor at the *Escola Bahiana de Medicina e Saúde Pública*, in Brazil, performed the largest number of studies on the subject (44 articles) during the period evaluated. Similarly, Casseb J (34 articles) and Ishak R (24 articles), who belong to the Institute of Tropical Medicine of *São Paulo* and the Institute of Biological Sciences of the Federal University of *Pará*, respectively, ranked second and third. The high scientific production of Brazilian authors is to be expected as Brazil is a highly endemic country for HTLV-1 and HTLV-2 [39], and it has the necessary resources and infrastructure for scientific activity. In this regard, Brazil spends 1.21% of the gross domestic product on development and research, being the country with the highest percentage of LAC spending on this item [40].

The journal with the most publications on HTLV-1 and HTLV-2 in LAC was *Aids Research and Human Retroviruses*, followed by the *Revista da Sociedade Brasileira de Medicina Tropical*, both with 29 articles each. The 10 journals with the most publications contained 39% of the total publications during the study period, being 8 Anglo-American and 2 Brazilian. Although the majority of LAC countries are Hispanic, except for Brazil, the high proportion of English-language publications may be attributed to the increasing accessibility of LAC authors to publish in prestigious journals, with language becoming a lesser barrier to scientific communication [41].

Brazil had the largest number of publications by correspondent authors in that country, with a total of 313 articles, representing 60% of all the articles evaluated. It should be noted that among the 10 countries with the highest number of publications by correspondent authors, half were from LAC and the other half from Europe and the United States. This result may indicate the diversity of collaboration networks between institutions in LAC and high-income countries [41]. Likewise, the institutions with the largest number of articles on HTLV-1 were the *Fundaçao Oswaldo Cruz Bahia*, the University of *São Paulo*, the Federal University of *Par*á*,* and the Federal University of *Bahia*, all of which are Brazilian. Although Brazilian institutions generate high-quality studies, the scientific impact is low due to the high degree of internal collaboration and the low magnitude of international collaborations [42]. This was evidenced in our analysis, since Brazil was the country with the highest number of SCP with 249 publications, representing 79.5% of its total production in the present analysis. In addition, it has been shown that collaborations between LAC countries and developed countries increase the impact factor of the articles, and, conversely, articles without collaborations, although published in prestigious journals, have a lower impact [43]. Therefore, the governments of LAC countries must promote research on HTLV-1 and HTLV-2, and ensure that it can be carried out in partnership with international institutions. Among the research topics, priority should be given to those that guide the formulation of public health interventions to reduce the transmission of the virus in the populations at greatest risk in LAC [15].

In 2015, Bangham CRM, together with Abelardo A (Brazilian author), Yoshihisa Y and Graham PT, published a literature review in the journal *Nature Reviews Disease Primers* on tropical spastic paraparesis [26], entitled “HTLV-1-associated myelopathy/tropical spastic paraparesis”, which is one of the conditions commonly associated with HTLV-1 infection. This article had the greatest impact on the scientific community in terms of publications on the subject, with 119 citations and an average of 14.9 citations per year from its publication in 2015 to date. The article addresses a review of various aspects such as epidemiology; biology, pathophysiological mechanisms, and diagnosis, screening, and prevention of tropical spastic paraparesis associated with HTLV-1. One of the conclusions of the document was that there is still a lack of evidence from basic and clinical research to identify therapeutic schemes for the clinical management of this disease. Likewise, the study proposes a series of knowledge gaps to guide future publications on the subject.

Among the most used keywords, most refer to the virology of HTLV-1 and its associated diseases (i.e. myelopathy, adult T-cell leukemia/lymphoma). Other keywords such as “Infections”, “Prevalence” and “Blood-donors”, were also common, as previously reported in other bibliometric analysis [20,36]. This is particularly true for Brazil, which seems to produce more prevalence-based studies, possibly because of its vast territory (Fig. 4). Of note was the low number of terms related to therapies, being only “antiviral therapy”, a small and less related term to HTLV-1, which coincides with the most cited study mentioned in the previous paragraph. However, although there are few novel treatments for HTLV-1 infection, there is high interest in the scientific community to implement clinical trials in special groups, such as preventive programs with antiretroviral therapy to avoid maternal vertical transmission [9,44]. Thus, as HTLV-1 endemicity in LAC provides potential candidates for studies, more incentives should be given to LAC researchers to develop high-quality control trials.

One of the main limitations of this study is that a bibliometric analysis depends on the availability of the data of the articles obtained with the search strategy. On the other hand, a single database (WOS) for the analysis, and the production of other databases, such as PubMed, Scopus, or regional databases, was not considered. However, the WOS database is considered to be one of the most complete to carry out this type of analysis, since it has records from the year 1900 and more than 34,000 journals indexed around the world [22].

## 5 Conclusions

In conclusion, according to the present quantitative analysis, there is a need for more significant promotion of research on HTLV-1 and HTLV-2 among the scientific community in LAC. We have synthesized and described the bibliometric indicators regarding this topic during the last 10 years, providing past and present guidance on this topic as a basis for future collaborations and research and a foundation for further research in LAC. Furthermore, the government bodies and agencies that regulate research and development in the different LAC countries must continue to promote and finance new research on this subject, which is of interest to global health. Finally, future studies could develop bibliometric analysis of research on HTLV-1 and HTLV-2 by separate.

## Author contribution statement

Akram Hernández-Vásquez: Conceived and designed the experiments; Analyzed and interpreted the data; Contributed reagents, materials, analysis tools or data; Wrote the paper.Fabriccio J. Visconti-Lopez: Contributed reagents, materials, analysis tools or data; Analyzed and interpreted the data; Wrote the paper.Dustin M. Solorzano-Salazar: Analyzed and interpreted the data; Wrote the paper.Diego Azañedo: Analyzed and interpreted the data; Wrote the paper.

## Funding statement

This research did not receive any specific grant from funding agencies in the public, commercial, or not-for-profit sectors.

## Data availability statement

Data included in article/supplementary material/referenced in article.

## Declaration of competing interest

The authors declare that they have no known competing financial interests or personal relationships that could have appeared to influence the work reported in this paper.
