# Supplementary material for: Research on HTLV-1 and HTLV-2 in Latin America and the Caribbean over the last ten years
Source: Heliyon. 2023 Feb 15;9(3):e13800. doi: 10.1016/j.heliyon.2023.e13800 (PMC9958499; doi:10.1016/j.heliyon.2023.e13800)
Supplement: Multimedia component 1 [file mmc1.docx]

| **Search** | **Web of Science Core Collection** |
| --- | --- |
| #19 | #17 AND #18 AND Article (Document Types) AND 2022 (Exclude – Publication Years) |
| #18 | TS=Latin America OR TI=(Latin NEAR/1 America*) OR AB=(Latin NEAR/1 America*) OR ALL=Latinamerica* OR ALL=Latinoamerica* OR ALL=Hispanoamerica* OR ALL=Iberoamerica* OR TI=(Ibero NEAR/1 America*) OR AB=(Ibero NEAR/1 America*) OR ALL=Panamerican* OR TS=Central America OR TI=(Central NEAR/1 America*) OR AB=(Central NEAR/1 America*) OR ALL=Centroamerica* OR ALL=Mesoamerica* OR TI=(Meso NEAR/1 America*) OR AB=(Meso NEAR/1 America*) OR TI=(Middle NEAR/1 America*) OR AB=(Middle NEAR/1 America*) OR TS=South America OR TI=(South NEAR/1 America*) OR AB=(South NEAR/1 America*) OR ALL=Southamerica* OR ALL=Sudamerica* OR TI=(America NEAR/2 Sur) OR AB=(America NEAR/2 Sur) OR TS=Caribbean Region OR ALL=Caribbean OR ALL=Caribe* OR TS=West Indies OR TI=(West NEAR/1 Indi*) OR AB=(West NEAR/1 Indi*) OR ALL=Antill* OR TS=Indians, South American OR TS=Indians, Central American OR ALL=Amerindian* OR TI=(America* NEAR/3 Indian*) OR AB=(America* NEAR/3 Indian*) OR TI=(Native NEAR/1 America*) OR AB=(Native NEAR/1 America*) OR ALL=Patagoni* OR ALL=Andes OR ALL=Andean* OR ALL=Amazon* OR ALL=Anguill* OR ALL=(“Antigua Barbud*”) OR ALL=Argentin* OR ALL=Aruba* OR ALL=Baham* OR ALL=Barbad* OR ALL=Belize* OR ALL=Bermud* OR ALL=Bolivia* OR ALL=Brazil* OR ALL=Brasil* OR ALL=Cayman* OR ALL=Chile* OR ALL=Colombia* OR TI=(“Costa Ric*”) OR ALL=(“Costa Ric*”) OR ALL=Costarric* OR ALL=Costaric* OR ALL=Cuba* OR ALL=Curaçao* OR ALL=Dominic* OR ALL= Ecuador* OR ALL=Salvador* OR ALL=(“Falkland Island*”) OR ALL=Guian* OR ALL=Grenad* OR ALL=Guadeloup* OR ALL=Guatemal* OR ALL=Guyan* OR ALL=Haiti* OR ALL=Hondur* OR ALL=Jamaic* OR ALL=Martiniqu* OR ALL=Martinic* OR ALL=Mexic* OR ALL=Montserrat* OR ALL=Antille* OR ALL=Nicaragu* OR ALL=Panam* OR ALL=Paraguay* OR ALL=Peru* OR ALL=(“Puerto Ric*”) OR ALL=Puertorric* OR ALL=(“Saint Kitts*”) OR ALL=Kittitian* OR ALL=Nevisian* OR ALL=(“Saint Luc*”) OR ALL=(“Saint Vincent*”) OR ALL=(“Sint Maarten*”) OR ALL=”South Georgia Island” OR ALL=”South Sandwich Island” OR ALL=Surinam* OR ALL=Tobago* OR ALL=Trinidad* OR ALL=“Turks and Caicos” OR ALL=Uruguay* OR ALL=Venez* OR ALL=(“Virgin Island*”) |
| #17 | #16 OR #15 OR #14 OR #13 OR #12 OR #11 OR #10 OR #9 OR #8 OR #7 OR #6 OR #5 OR #4 OR #3 OR #2 OR #1 |
| #16 | TI=(Leukemia NEAR/3 Virus-2) OR AB=(Leukemia NEAR/3 Virus-2) |
| #15 | TI=(Leukemia NEAR/3 Virus-II) OR AB=(Leukemia NEAR/3 Virus-II) |
| #14 | TI=HTLV-2 OR AB=HTLV-2 |
| #13 | TI=HTLV-II OR AB=HTLV-II |
| #12 | TI=(T-Lymphotropic NEAR/3 Virus-II) OR AB=(T-Lymphotropic NEAR/3 Virus-II) |
| #11 | TI=(T-Lymphotropic NEAR/3 Virus-2) OR AB=(T-Lymphotropic NEAR/3 Virus-2) |
| #10 | TS=Human T-Lymphotropic Virus 2 |
| #9 | TI=(Leukemia NEAR/3 Virus-1) OR AB=(Leukemia NEAR/3 Virus-1) |
| #8 | TI=(Leukemia NEAR/3 Virus-I) OR AB=(Leukemia NEAR/3 Virus-I) |
| #7 | TS=Human T-lymphotropic virus |
| #6 | TS=HTLV-II Infections |
| #5 | TI=(T-Lymphotropic NEAR/3 Virus-I) OR AB=(T-Lymphotropic NEAR/3 Virus-I) |
| #4 | TI=(T-Lymphotropic NEAR/3 Virus-1) OR AB=(T-Lymphotropic NEAR/3 Virus-1) |
| #3 | TI=HTLV-I OR AB=HTLV-I |
| #2 | TI=HTLV-1 OR AB=HTLV-1 |
| #1 | TS=HTLV-I Infections |

**Search strategy for included articles (n = 519)**

UT=(WOS:000466360100016 OR WOS:000329981700008 OR WOS:000311884500001 OR WOS:000353695000013 OR WOS:000610104700016 OR WOS:000579365500009 OR WOS:000468789200001 OR WOS:000308337300022 OR WOS:000417587300002 OR WOS:000489819200020 OR WOS:000434099300004 OR WOS:000683353600028 OR WOS:000672556800001 OR WOS:000474572700001 OR WOS:000311106100011 OR WOS:000418613300006 OR WOS:000308206000096 OR WOS:000376656800014 OR WOS:000499613500004 OR WOS:000311981100020 OR WOS:000373627000019 OR WOS:000357305800001 OR WOS:000558076200028 OR WOS:000431268900035 OR WOS:000330899200007 OR WOS:000428210500006 OR WOS:000636737700026 OR WOS:000371080700011 OR WOS:000373599300002 OR WOS:000764676900001 OR WOS:000554403900001 OR WOS:000364109900010 OR WOS:000456711500008 OR WOS:000404453600013 OR WOS:000489762300034 OR WOS:000719589200024 OR WOS:000514146800001 OR WOS:000324481600109 OR WOS:000407039200011 OR WOS:000311825000035 OR WOS:000383657100001 OR WOS:000400644400009 OR WOS:000322992100018 OR WOS:000531085300034 OR WOS:000303209600004 OR WOS:000398708500015 OR WOS:000441751200013 OR WOS:000585290800001 OR WOS:000298383500009 OR WOS:000631260500023 OR WOS:000537280000049 OR WOS:000393895000024 OR WOS:000412751100006 OR WOS:000311262000007 OR WOS:000397680700002 OR WOS:000384630100020 OR WOS:000488520700024 OR WOS:000661574500033 OR WOS:000581379500001 OR WOS:000309329700015 OR WOS:000398739500005 OR WOS:000485006800051 OR WOS:000368676100014 OR WOS:000381307700001 OR WOS:000651872600009 OR WOS:000443313800009 OR WOS:000300411500016 OR WOS:000384630100012 OR WOS:000684605900012 OR WOS:000636246400001 OR WOS:000417695200024 OR WOS:000330319500004 OR WOS:000311124000001 OR WOS:000318072700014 OR WOS:000554606400014 OR WOS:000493891000005 OR WOS:000655321700010 OR WOS:000317345300006 OR WOS:000367733900025 OR WOS:000320605400050 OR WOS:000633999700001 OR WOS:000413606800058 OR WOS:000505473500030 OR WOS:000362104000006 OR WOS:000313348900011 OR WOS:000354972200041 OR WOS:000431525100001 OR WOS:000441196300005 OR WOS:000574836900002 OR WOS:000330319500017 OR WOS:000402328700006 OR WOS:000340575100021 OR WOS:000336075000005 OR WOS:000563938300028 OR WOS:000693852800001 OR WOS:000705554300001 OR WOS:000304658000001 OR WOS:000708782800002 OR WOS:000419880700002 OR WOS:000330429800015 OR WOS:000569065400004 OR WOS:000300696200016 OR WOS:000413881300001 OR WOS:000417493500021 OR WOS:000345430900019 OR WOS:000335900000042 OR WOS:000319253800018 OR WOS:000461315600001 OR WOS:000367158500017 OR WOS:000462938900008 OR WOS:000399949900015 OR WOS:000495408600004 OR WOS:000326129900027 OR WOS:000435797200019 OR WOS:000383857500015 OR WOS:000302121700020 OR WOS:000424572800009 OR WOS:000314914200007 OR WOS:000329818300005 OR WOS:000413876500009 OR WOS:000645061300006 OR WOS:000346237700010 OR WOS:000364260500007 OR WOS:000320378300005 OR WOS:000341335200006 OR WOS:000348183200001 OR WOS:000341834400009 OR WOS:000443206900007 OR WOS:000614371400001 OR WOS:000394483800003 OR WOS:000305945300031 OR WOS:000424022500003 OR WOS:000401767400021 OR WOS:000474278400001 OR WOS:000365625000003 OR WOS:000299137300013 OR WOS:000340790000001 OR WOS:000432831700001 OR WOS:000417193500017 OR WOS:000385723000018 OR WOS:000209928700015 OR WOS:000305980500005 OR WOS:000397584200015 OR WOS:000341526800007 OR WOS:000568698400009 OR WOS:000455207700027 OR WOS:000309631200024 OR WOS:000315587900001 OR WOS:000321903400008 OR WOS:000393496600001 OR WOS:000369711400014 OR WOS:000329979600013 OR WOS:000371322400018 OR WOS:000308943900020 OR WOS:000553123400002 OR WOS:000620940200001 OR WOS:000396161700056 OR WOS:000305980500006 OR WOS:000406170700015 OR WOS:000303264600009 OR WOS:000321201300029 OR WOS:000373465500009 OR WOS:000312794500058 OR WOS:000427901500022 OR WOS:000321119300008 OR WOS:000306648800014 OR WOS:000356317400017 OR WOS:000355378500013 OR WOS:000539447400001 OR WOS:000672492300001 OR WOS:000317592400007 OR WOS:000323372600004 OR WOS:000349144400125 OR WOS:000634742200001 OR WOS:000469224300050 OR WOS:000395461600015 OR WOS:000461889700062 OR WOS:000324498000009 OR WOS:000582325600034 OR WOS:000608403200001 OR WOS:000533498800025 OR WOS:000307527400008 OR WOS:000482379500001 OR WOS:000513130300002 OR WOS:000705008200004 OR WOS:000602463800001 OR WOS:000319625400024 OR WOS:000306894200005 OR WOS:000382306500023 OR WOS:000394667800038 OR WOS:000673505300005 OR WOS:000426933700019 OR WOS:000388574400055 OR WOS:000342361000017 OR WOS:000373527100017 OR WOS:000333038700016 OR WOS:000393895000020 OR WOS:000386872100026 OR WOS:000724926900007 OR WOS:000305981400011 OR WOS:000305945300044 OR WOS:000346701000056 OR WOS:000498961500001 OR WOS:000475498800010 OR WOS:000697112000006 OR WOS:000515930700082 OR WOS:000477099300001 OR WOS:000624570900012 OR WOS:000477065200001 OR WOS:000312680400014 OR WOS:000456009100010 OR WOS:000399371900161 OR WOS:000460896500001 OR WOS:000341526800013 OR WOS:000317238300013 OR WOS:000677374500001 OR WOS:000408247100019 OR WOS:000312112900015 OR WOS:000304546500002 OR WOS:000419170300001 OR WOS:000399371900051 OR WOS:000417861700009 OR WOS:000408287600030 OR WOS:000311060000027 OR WOS:000498804500001 OR WOS:000322321500038 OR WOS:000614768500005 OR WOS:000330383800056 OR WOS:000405242000024 OR WOS:000724777300001 OR WOS:000321949400011 OR WOS:000360254900004 OR WOS:000489611000001 OR WOS:000682920500001 OR WOS:000307840800010 OR WOS:000324750000029 OR WOS:000329045500007 OR WOS:000760118200024 OR WOS:000403190300004 OR WOS:000473779100026 OR WOS:000405320700008 OR WOS:000451308900009 OR WOS:000427254600029 OR WOS:000386875300013 OR WOS:000394071300022 OR WOS:000531144000001 OR WOS:000367848600001 OR WOS:000310356400059 OR WOS:000353073800004 OR WOS:000344443500004 OR WOS:000364916400001 OR WOS:000389671800001 OR WOS:000334868600002 OR WOS:000477072100001 OR WOS:000452162500037 OR WOS:000575752100002 OR WOS:000486053500005 OR WOS:000312417200010 OR WOS:000329291200016 OR WOS:000298060000020 OR WOS:000449318100044 OR WOS:000468256000006 OR WOS:000467856200001 OR WOS:000355185600111 OR WOS:000414340200011 OR WOS:000318074200013 OR WOS:000533889300045 OR WOS:000301160200021 OR WOS:000470209500009 OR WOS:000417628500013 OR WOS:000321699700013 OR WOS:000535214700005 OR WOS:000486055000004 OR WOS:000397963900003 OR WOS:000340294800007 OR WOS:000310734800001 OR WOS:000726192000001 OR WOS:000339706500015 OR WOS:000302889200004 OR WOS:000307693600002 OR WOS:000591891900001 OR WOS:000487823900003 OR WOS:000308959600007 OR WOS:000343815700001 OR WOS:000315323000034 OR WOS:000306355500009 OR WOS:000368040300012 OR WOS:000428386900003 OR WOS:000559317800007 OR WOS:000342796600035 OR WOS:000310532000014 OR WOS:000341473600012 OR WOS:000468304300003 OR WOS:000486051500004 OR WOS:000399352000023 OR WOS:000372694700074 OR WOS:000475399900027 OR WOS:000378595100037 OR WOS:000382506100015 OR WOS:000605548100004 OR WOS:000369184000022 OR WOS:000435228000016 OR WOS:000302843100007 OR WOS:000727813000014 OR WOS:000362636600001 OR WOS:000432339100005 OR WOS:000317995200007 OR WOS:000450995200002 OR WOS:000312944600012 OR WOS:000413876500008 OR WOS:000606454900001 OR WOS:000432390600014 OR WOS:000365871300004 OR WOS:000396381700005 OR WOS:000675812400008 OR WOS:000505687400004 OR WOS:000718236200002 OR WOS:000304541000012 OR WOS:000558074700070 OR WOS:000705281200002 OR WOS:000342361000023 OR WOS:000340488700008 OR WOS:000443381000050 OR WOS:000477251200001 OR WOS:000374832500013 OR WOS:000308748400002 OR WOS:000351308400005 OR WOS:000330378400029 OR WOS:000360708200049 OR WOS:000397947500021 OR WOS:000437026000016 OR WOS:000365651700020 OR WOS:000308100800007 OR WOS:000330829200058 OR WOS:000363756400007 OR WOS:000647335400017 OR WOS:000553994500001 OR WOS:000470781100001 OR WOS:000365656000001 OR WOS:000663853400001 OR WOS:000470188100001 OR WOS:000518442900008 OR WOS:000429122000021 OR WOS:000325639400013 OR WOS:000321735900009 OR WOS:000369265600001 OR WOS:000300583000013 OR WOS:000658832600019 OR WOS:000480544400003 OR WOS:000309050400001 OR WOS:000306683600011 OR WOS:000342796600067 OR WOS:000302889200005 OR WOS:000560547200001 OR WOS:000471238000022 OR WOS:000617988100001 OR WOS:000445572100013 OR WOS:000316721300012 OR WOS:000393915400001 OR WOS:000333034900012 OR WOS:000383669500022 OR WOS:000571668800001 OR WOS:000330094100021 OR WOS:000528164100035 OR WOS:000568201700001 OR WOS:000613976000001 OR WOS:000320166500006 OR WOS:000540480900001 OR WOS:000551523900031 OR WOS:000314097700001 OR WOS:000687567500004 OR WOS:000461522500028 OR WOS:000394813700001 OR WOS:000345515800010 OR WOS:000568698400007 OR WOS:000323372600015 OR WOS:000410909200021 OR WOS:000515694700001 OR WOS:000428112700005 OR WOS:000323564500056 OR WOS:000322929700006 OR WOS:000717022400001 OR WOS:000367590100008 OR WOS:000334649200013 OR WOS:000541047600002 OR WOS:000442171900024 OR WOS:000301160200011 OR WOS:000325910600006 OR WOS:000333236600001 OR WOS:000307627800013 OR WOS:000359323500009 OR WOS:000581741700005 OR WOS:000512881500026 OR WOS:000422853400013 OR WOS:000436264900005 OR WOS:000629400900009 OR WOS:000402462200012 OR WOS:000559370500025 OR WOS:000325517000007 OR WOS:000328212900018 OR WOS:000344332300013 OR WOS:000432583100001 OR WOS:000452400500039 OR WOS:000479030000028 OR WOS:000401879000037 OR WOS:000337224400015 OR WOS:000482092100063 OR WOS:000579359800001 OR WOS:000515089200002 OR WOS:000327726800025 OR WOS:000302889200006 OR WOS:000325190400047 OR WOS:000432673400001 OR WOS:000350791900001 OR WOS:000375072600004 OR WOS:000330319500006 OR WOS:000319183800022 OR WOS:000402035800001 OR WOS:000452571700001 OR WOS:000404453600003 OR WOS:000316300400001 OR WOS:000614083100017 OR WOS:000473779100024 OR WOS:000323359800010 OR WOS:000474999200001 OR WOS:000380971100012 OR WOS:000588422900059 OR WOS:000332281200005 OR WOS:000448786200017 OR WOS:000307101900022 OR WOS:000460698300016 OR WOS:000419944200015 OR WOS:000315644900006 OR WOS:000571454400003 OR WOS:000600757900003 OR WOS:000457773000018 OR WOS:000627781200001 OR WOS:000367335100010 OR WOS:000386983800022 OR WOS:000337486800011 OR WOS:000596080300006 OR WOS:000395011300011 OR WOS:000313565100016 OR WOS:000425575400009 OR WOS:000329291200015 OR WOS:000445907400051 OR WOS:000553858800074 OR WOS:000309066800015 OR WOS:000387657500024 OR WOS:000443682600038 OR WOS:000340842300010 OR WOS:000500684400001 OR WOS:000442468400005 OR WOS:000354880200014 OR WOS:000450904400009 OR WOS:000371812500010 OR WOS:000404095900019 OR WOS:000398647900006 OR WOS:000660899200002 OR WOS:000593122700037 OR WOS:000354912400006 OR WOS:000510866400012 OR WOS:000335970300005 OR WOS:000430504300027 OR WOS:000468944300011 OR WOS:000394831200020 OR WOS:000724967400001 OR WOS:000554780500002 OR WOS:000355785400029 OR WOS:000318976300017 OR WOS:000448919000001 OR WOS:000605329000010 OR WOS:000443206900001 OR WOS:000438457400074 OR WOS:000366540500011 OR WOS:000449867400009 OR WOS:000661464700137 OR WOS:000344812500047 OR WOS:000535954500001 OR WOS:000368695700013 OR WOS:000346072500005 OR WOS:000434755500012 OR WOS:000455854900004 OR WOS:000307627800049 OR WOS:000372683500013 OR WOS:000374343300010 OR WOS:000523462600012 OR WOS:000360519100001 OR WOS:000383336100010 OR WOS:000456709700041 OR WOS:000351659200040 OR WOS:000349318100019 OR WOS:000324915800013 OR WOS:000412179600025 OR WOS:000312672800008 OR WOS:000577244600009 OR WOS:000313565100009 OR WOS:000590969600001 OR WOS:000331125500041 OR WOS:000324750000032 OR WOS:000300193600033 OR WOS:000388574000004 OR WOS:000443206900005 OR WOS:000606166800001 OR WOS:000326176400017 OR WOS:000381899000031 OR WOS:000548754300001 OR WOS:000347633800031 OR WOS:000309011200015 OR WOS:000719817900006 OR WOS:000305839600001 OR WOS:000312183900053 OR WOS:000563550700002 OR WOS:000565262300001 OR WOS:000447817100017 OR WOS:000443419200001 OR WOS:000346237700004 OR WOS:000488807700001 OR WOS:000463120800011)
